# Provenance effect on the fine-root metabolome and structure of mature Scots pine revealed in a long-term common garden experiment

**DOI:** 10.1093/treephys/tpag126

**Published:** 2026-09-08

**Authors:** Agnieszka Szuba, Pulak Maitra, Katarzyna Hrynkiewicz, Izabela Ratajczak, Daniel J Chmura, Joanna Mucha

**Affiliations:** Department of Genetics and Environmental Interactions, Institute of Dendrology Polish Academy of Sciences, Parkowa 5, PL62035 Kornik, Poland; Department of Ecology, Institute of Dendrology Polish Academy of Sciences, Parkowa 5, PL62035 Kornik, Poland; W. K. Kellogg Biological Station, Michigan State University, 3700 East Gull Lake Drive, Hickory Corners, MI 49060, USA; Department of Microbiology, Faculty of Biological and Veterinary Sciences, Nicolaus Copernicus University in Toruń, Lwowska 1, PL87100 Toruń, Poland; Department of Chemistry, Poznań University of Life Sciences, Wojska Polskiego 75, Poznań, PL60625, Poland; Department of Genetics and Environmental Interactions, Institute of Dendrology Polish Academy of Sciences, Parkowa 5, PL62035 Kornik, Poland; Department of Ecology, Institute of Dendrology Polish Academy of Sciences, Parkowa 5, PL62035 Kornik, Poland

**Keywords:** adult trees, amino acids, carbohydrates, common garden experiment, GC MS/MS, in situ study, provenances, root morphology

## Abstract

Rapid changes in temperature and precipitation present a major challenge for long-lived forest trees, as their long generation times limit the required rate of adaptive responses within populations. Common garden experiments provide valuable insights into local adaptation, but belowground responses remain understudied. We investigated fine root metabolomes, biochemistry and structure in 54-year-old *Pinus sylvestris* L. originating from two contrasting environments: a local lowland site and a colder high-elevation mountain region, both grown in a common garden. Untargeted gas chromatography-tandem mass spectrometry profiling identified 160 metabolites and revealed pronounced effects of both season and tree origin. In spring, roots from high-elevation trees exhibited a distinct metabolomic profile characterized by lower concentrations of nitrogen-containing compounds and elevated levels of lipid derivatives, including fatty acids and sterols. In contrast, autumn roots from both origins showed broadly similar metabolite abundances. According to biochemical analyses, seasonal shifts were also evident in carbohydrate dynamics, with higher concentrations of non-structural carbohydrates in spring and increased structural carbohydrates deposition in autumn. Nitrogen compounds, particularly amino acids, increased in autumn roots of both tree origins, most likely reflecting nutrient storage. Differences related to tree origin extended to root morphology, as roots from high-elevation trees had higher cell wall carbohydrate content and altered root branching patterns compared with those from lowland trees. Our findings suggest that tree origin leaves a clear imprint on the fine root metabolome and structure of mature Scots pine, with most pronounced differences occurring in early spring. These results highlight how inherited adaptation to origin climates interacts with seasonal soil processes. High-resolution metabolomics thus offers a powerful tool for detecting subtle belowground provenance-related belowground effects that may be overlooked by traditional trait-based approaches, providing new perspectives on possible tree adaptation under climate change.

## Graphical Abstract

**Graphical Abstract:**
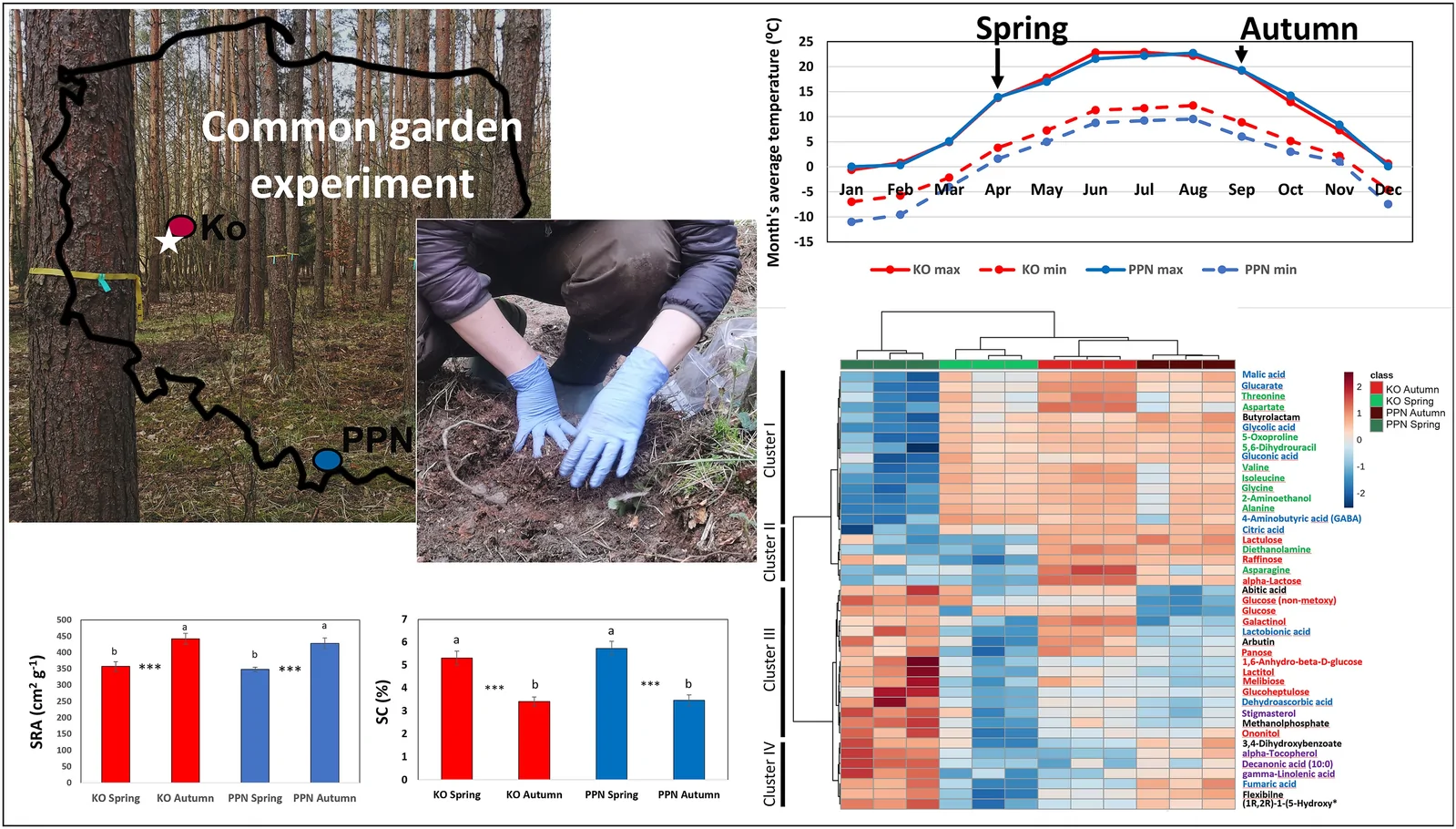

## Introduction

Rapid and expected shifts in air temperature and precipitation patterns (IPCC 2024) present significant challenges for long-lived plant species such as trees. Trees are sessile organisms that must endure environmental changes within their existing habitats (Aubin et al. 2016). The projected pace of climate change may likely exceed the ability of tree species’ populations to adapt in situ (Martin et al. 2023). Common garden experiments are invaluable for studying local adaptation, as they enable researchers to evaluate both individual traits and the combined expression of multiple traits across broad climatic gradients (Cooper et al. 2019, Germino et al. 2019). While much focus in common garden studies has been placed on adaptive variation in growth and aboveground traits of trees, studies on belowground traits’ variation are still comparatively rare (Laliberté 2017). Examining root traits is essential for gaining a more complete understanding of plant functional strategies, especially regarding nutrient acquisition and biotic interactions that cannot be easily inferred from aboveground traits alone (Carmona et al. 2021, Weemstra et al. 2022). Despite the important ecological roles of root systems, research specifically addressing Scots pine root adaptation to changing climate conditions through common garden studies remains limited (Zadworny et al. 2017, 2021).

Plants persist under changing environmental conditions through phenotypic plasticity and local adaptation (Šurinová et al. 2019). Widely distributed species still show substantial variation among provenances (Münzbergová et al. 2017). However, the reasons for the differences among provenances still remain unclear, especially in mature trees and belowground traits. Common garden experiments distinguish environmental from genetic effects. In Scots pine, provenance differences are strongest in juveniles, while environmental effects dominate in mature trees (Jankowski et al. 2019). Under rapid environmental change, locally adapted populations may lag behind shifting conditions (Rehfeldt et al. 2002, Mátyás et al. 2023), and long lifespans further constrain adaptation, particularly at colder margins (Aitken et al. 2008).

Root metabolic processes are central to these adaptive responses (Hunziker et al. 2024). Soil temperature strongly influences root growth in temperate forests (Alvarez-Uria and Körner 2007), and fine root development depends on non-structural carbohydrates (NSCs) such as fructose, sucrose, glucose and starch (Hoch et al. 2003, Wang et al. 2018). Non-structural carbohydrates maintain root health during short growing seasons and support nitrogen absorption through interactions with ectomycorrhizal fungi (Rosling et al. 2008). Root growth and cold tolerance are closely linked to nitrogen, carbon and lipid metabolism (Gabukova et al. 1991, Toca et al. 2018), while root-associated microbiota are further regulated by nitrogen cycling and carbohydrate dynamics (Yuan et al. 2023, Maitra et al. 2024*a*). Together, these processes drive seasonal changes in root metabolomes and likely underlie provenance-level variation in adaptive responses.

Seasonal asynchrony between above- and belowground activity is especially pronounced in spring, when air warms faster than soil, delaying root activity relative to photosynthesis (Wu et al. 2011). Seasonal carbohydrate cycling is vital for root respiration, cold tolerance and early growth (Wong et al. 2009, Bazot et al. 2013), with NSC concentrations typically peaking in late summer and declining through dormancy (Barbaroux and Bréda 2002, Furze et al. 2018). Provenance trials have revealed differences in soil activity and biomass between Scots pine populations from northern and southern Europe (Kieliszewska-Rokicka et al. 2003), suggesting that carbon allocation strategies are shaped by seed origin. These findings highlight the need to characterize seasonal metabolic variation in fine roots of Scots pine provenances originating from contrasting climates.

Scots pine (*Pinus sylvestris* L.) is one of the most widely distributed forest tree species, with a range spanning diverse climatic and edaphic conditions (Boratyński 1993). Such diversity has driven both acclimation and local adaptation of populations to various climatic and soil conditions (Bose et al. 2020, 2024, Żukowska et al. 2023). To better understand metabolic processes related to the acclimatory response of plants transferred to warmer environments, we used a common garden study with 54-year-old Scots pine, analyzing fine root metabolome (<2 mm diameter) of two provenances: a local ecotype and a provenance originating from higher elevation and cooler climate (mean annual temperature, Δ MAT ~ 2 °C, total annual precipitation, Δ MAP ~ 170 mm year^−1^). Because all trees were grown in a single common garden environment, our study focuses on environmentally driven seasonal responses and inherited differences among tree origins rather than directly quantifying phenotypic plasticity across contrasting environments. We utilize gas chromatography-tandem mass spectrometry (GC–MS/MS)-based profiling, as metabolomics provides a powerful and sensitive approach for investigating physiological variation in mature trees (Hunziker et al. 2024).

We hypothesized that genetic adaptation to the local environments of the provenances would result in distinct metabolomic signatures in the fine roots of mature Scots pine trees of two provenances grown under common garden conditions. More specifically, we expected that (i) root metabolomes of the two provenances would remain distinguishable after more than 50 years of growth under the same environmental conditions, reflecting persistent provenance-related differences; (ii) provenance-related metabolic differences would involve pathways associated with carbon metabolism, nitrogen metabolism, membrane lipid metabolism and stress-related compounds, which are known to contribute to plant responses to environmental variation; and (iii) seasonal conditions would modify the extent and composition of provenance-related metabolic differences between spring and autumn.

## Materials and methods

### Study site and plant material

The analyzed roots were collected from adult trees growing as part of a large common garden experiment established in 1967, in the Experimental Forest ‘Zwierzyniec’ of the Institute of Dendrology of the Polish Academy of Sciences in Kórnik (Przybylski 1970; all information regarding the design of this experiment is presented in File S1 available as Supplementary Data at *Tree Physiology* Online). The experiment is located on a lowland area with flat relief on former agricultural land. The soils are classified as Luvisols, developed from sandy and sandy–loamy deposits (sands and sandy loams), which are distinguished by moderate fertility and excellent permeability. These site characteristics suggest relatively low edaphic heterogeneity across the plot, although small-scale microsite variation cannot be excluded.

Based on our previous studies, from 31 provenances grown in the analyzed common garden (File S1 available as Supplementary Data at *Tree Physiology* Online), for the current investigation we have chosen two provenances: Pieniński Park Narodowy (PPN) and a local one from Kórnik (KO) (Figure 1a). The trial was designed for provenance-level comparisons; therefore, cones were collected from at least 10 randomly selected trees per provenance and seeds were bulked before sowing. Consequently, mother-tree identity and family structure were unavailable for the present study. The KO seed origin was the warmest and driest and the high-elevation mountain PPN origin region was the coldest and wettest among tested provenances. The PPN provenance had also more indicator species of microorganisms (fungi and bacteria) and higher carbohydrate concentrations in fine roots (<2 mm) than other studied provenances (Maitra et al. 2024*b*).

**Figure 1:**
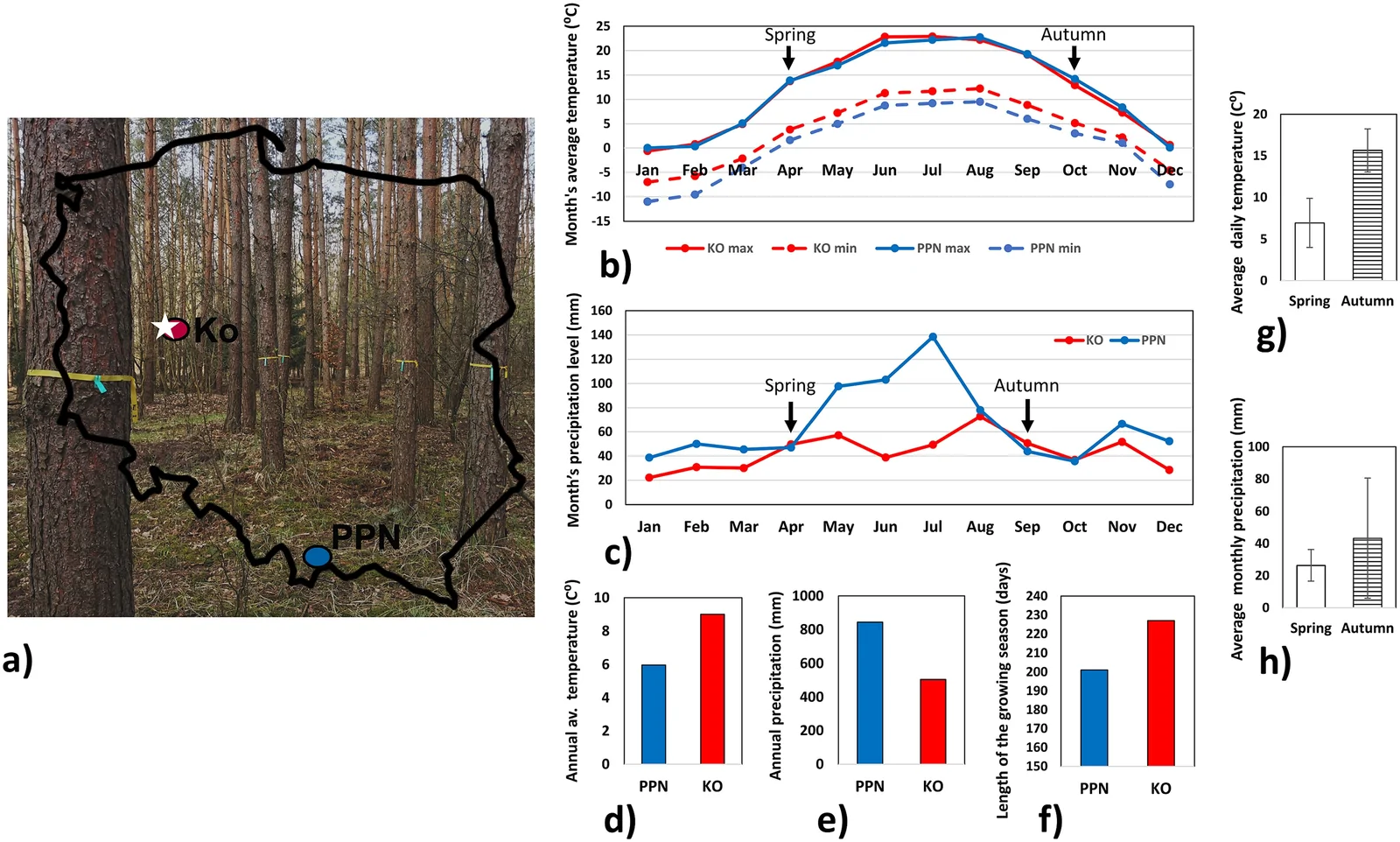
Experiment overview. The representative view of the common garden experiment plot in the Zwierzyniec Forest at the moment of spring harvesting with graph of location of the provenance origin marked on the map of Poland. Asterisk—the common garden site (a). Historical average month maximum and minimum temperatures observed for the locations of the seed origin (1959–64) covering the period of seed and cones formation and representing the climate conditions at the time of common garden establishment (the time points of the plant material harvestings as well as time points where the threshold of 5 °C was exceeded for PPN and KO in spring have been marked with arrows) (b). Historical average month precipitation levels observed for the locations of the seed origin (1959–64) covering the period of seed and cones formation and representing the climate conditions at the time of common garden establishment (the time points of the plant material harvestings have been marked with arrows) (c). Historical (see panel (a)) annual average temperature (d). Historical annual participation level (e). Average length of the growth season annotated for years 1959–70 (f). Average daily temperature for harvesting months (±SD) for study site (g) average monthly precipitation calculated for 3 months preceding root harvesting points (±SD) for study site (h).

At the beginning of the common garden experiment, the survival percentage was assessed in 1971 and 1991 for 6- and 26-year-old trees, respectively, and was lower each time for the PPN than for KO provenance; that is, it was estimated at 95.2 and 21.5% for KO and at 88.3 and 12.5% for PPN, respectively (Przybylski 1970; File S1 available as Supplementary Data at *Tree Physiology* Online). In later years, thinnings were made, inhibiting the further reliable analyses of survival rates.

### Climatic variables for the seed origins and planting site

Given that climatic conditions up to 3 years before cone development influence seed formation in Scots pine (Philipson 1997), we obtained historical data from the 1959–64 period on minimum and maximum air temperatures (Figure 1b) and monthly precipitation (Figure 1c) as well as annual average values (Figure 1d and e, respectively) and length of the growth season (Figure 1f) from the weather stations relevant for provenances (https://www.imgw.pl/). Weather data for the 2021 study (for the common garden site) were obtained from a nearby station (Figure 1). To describe the long-term climate of seed origins and planting site, as well as the level of climate change throughout the duration of the common-garden experiment, we obtained detailed climatic data from the ClimateEU v.4.63 (Marchi et al. 2020) for provenances and planting site for a period 1941–70, corresponding to climate to which seed sources were adapted and the time of site establishment, and for 1991–2020 for planting site, corresponding to recent climate (Table S1 available as Supplementary Data at *Tree Physiology* Online).

### Sample collection

Roots of representative specimens were collected in late April (spring) and late September (autumn) of the same growing season in 2021. The same trees, located in at least four different blocks, were sampled in spring and autumn. In spring, during the plant material collection, the temperature was lower compared with autumn temperature (Figure 1g) whereas the precipitation levels (Figure 1h) were similar for both harvest points.

To reduce the influence of microsite heterogeneity, fine-root samples were collected from multiple trees within each provenance and across different blocks. For metabolomic and biochemical analyses, each biological replicate consisted of an independent pool of fine roots from at least five randomly selected trees collected from different positions within the experimental plot. This pooling strategy was intended to capture a provenance-level metabolic signal while reducing local edaphic noise (Szuba and Lorenc-Plucińska 2015); however, it does not allow estimation of individual-tree or family-level variance. Thus, each replicate integrated multiple trees and microsites, reducing—but not eliminating—the influence of localized soil variation. The weather conditions that influenced trees during the plant material harvesting (in Zwierzyniec Forest) were similar to historical climatic variables of KO (Figure 1g and h vs Table S1 available as Supplementary Data at *Tree Physiology* Online).

The sampled roots were transported in moist condition to the laboratory (for details see Maitra et al. 2024*b*) for further processing and analysis. The exception were the fine roots for metabolome study, which have been cleaned from soil particles in ice-cold miliQ water immediately after harvesting, carefully dried, frozen in liquid nitrogen in situ and next stored in −80 °C until analyzed.

For morphological analysis each of the seed origin was represented by 10–16 trees (~30 root lateral branches larger than 2 mm in diameter). For biochemical and metabolomic study of fine roots (with diameter ≤2 mm), each provenance was represented by 10–12 trees. Each sample represents randomly selected fine roots’ fragments of minimum five trees.

### Root morphology

After careful cleaning from soil particles in water, roots were subjected to measurements of morphological and biochemical parameters. Subsamples of roots were scanned with an Epson Perfection 3200 PHOTO scanner and the images were analyzed with the use of WinRhizo (Regent Instruments Inc.) image software to determine the morphological characteristics of each root system. The scanned root subsamples were then dried for 3 days at 65 °C and subsequently weighed. Specific root length (m g^−1^) was calculated as the ratio of total root length to root dry mass. Root tissue density (RTD, g cm^3^) was calculated by root dry mass by root volume.

### Root biochemistry

Dried fragments of fine roots were ground in a Retsch MM 200 mill (Retsch, Haan, Germany) and the resulting powder was used in further analyses. Concentrations of carbon and nitrogen were determined using the Elemental Combustion System CHNS-O 4010 (Costech Instruments, Italy/USA) using 2,5-(Bis(5-tert-butyl-2-benzo-oxazol-2-yl) thiophene for calibration. The protocol described by Oleksyn et al. (2000) was used to estimate the concentration of NSCs (total non-structural carbohydrates (TNC)—total soluble carbohydrates and starch). Sugars were extracted from dried roots with methanol–chloroform–water (12:5:3 by volume) (Avantor Performance Materials, Poland). To measure the level of soluble carbohydrates in the extract, anthrone reagent (Sigma) was added to the extract and the mixture was placed in a water bath (100 °C) for 12 min. Absorbance (625 nm) of the anthrone/extract mixture was measured within 30 min (Oleksyn et al. 2000). The insoluble material was converted to glucose by the addition of amyloglucosidase (15 U mL^−1^) (Sigma, St Louis, MO, USA) and incubating the mixture for 24 h at 50 °C. The peroxidase–glucose oxidase-o-dianisidine dihydrochloride reagent (Sigma) was used to determine glucose concentration and absorbance at 450 nm was measured within 30 min. of incubation at 25 °C. Concentrations of soluble sugars and starch (with glucose as a standard) are expressed as a percentage of dry mass.

The protein content was estimated using the Bradford method in protein extracts obtained by a modified phenolic extraction method (Szuba et al. 2020) from 50 mg of root powder (*n* = 6).

Total soluble phenolics in roots were determined using a colorimetric method described by Johnson and Schaal. Phenolic compounds were extracted with ethanol from dried root powder (*n* = 6), reacted with Folin–Ciocalteu reagent in Na₂CO₃ and quantified spectrophotometrically at 660 nm. The results were expressed as μmol chlorogenic acid equivalents per g dry weight.

H₂O₂ content was determined following the method of Zhou et al. (2006). Fine roots (100 mg; *n* = 6) were homogenized at 4 °C with activated carbon and 5% trichloroacetic acid (TCA) in a ratio of 1:0.2:1 (w/w/v). Hydrogen peroxide concentration was measured using a colorimetric reagent prepared by mixing 0.6 mM 4-(2-pyridylazo) resorcinol disodium salt with 0.6 mM potassium titanium oxalate (1:1) in 50 mM phosphate buffer (pH 8.4). Absorbance was recorded at 508 nm. H₂O₂ concentration was calculated from a standard curve and expressed as μmol g^−1^ fresh weight (FW).

Lipid peroxidation in *P. sylvestris* roots was assessed by determining malondialdehyde (MDA) content in frozen root powder homogenized in 0.25 M H₂SO₄/10% TCA according to a modified protocol of Shah et al. (2001) Thiobarbituric acid was added to the extract (0.25% v/v), and the mixture was boiled for 10 min. The reaction was stopped by cooling on ice. Absorbance of the supernatant was measured at 532 nm, with nonspecific absorbance corrected at 600 nm, and results were expressed per g FW as described by Stobrawa and Lorenc-Plucińska (2008).

Proline content was determined according to a modified protocol of Carillo and Gibon (2011). Frozen root powder (50 mg; *n* = 6) was extracted overnight in ethanol:water (40:60, v/v). The supernatant was then reacted with 1% ninhydrin prepared in 60% acetic acid and 20% ethanol. Absorbance was measured at 440 nm, and proline concentration was calculated from a standard curve and expressed as μM g^−1^ FW***.***

### FTIR spectroscopy analysis

Dried fine roots were ground in a Retsch MM 200 mill (Retsch, Haan, Germany). The resulting powder was mixed in the mill with potassium bromide (KBr) at a ratio of 1/200 mg, and the KBr pellets were made. Spectral analysis was conducted using a Nicolet iS5 spectrophotometer (Thermo Fisher Scientific, Waltham, MA, USA), recording 32 scans with a spectral resolution of 2 cm^−1^ in the range of 4000–500 cm^−1^, and each sample underwent 16 scans for data collection. FTIR analyses were conducted for the basic spectra to determine the presence of lignin, cellulose and hemicellulose in the analyzed root samples (Szuba et al. 2020). The combined spectra (*n* = 3) have been presented.

### Roots metabolome analysis

The frozen fine root samples were initially crushed in a mortar in liquid nitrogen and next powdered in a ball mill under liquid nitrogen. Metabolites were isolated from 200 mg pooled samples of roots (*n* = 3) by methanol extraction (Szuba et al. 2020). Following solvent evaporation, the dried extracts were subjected to a two-step derivatization procedure. Initially, the samples were incubated with 25 μL of methoxyamine hydrochloride solution in pyridine (20 mg mL^−1^) at 37 °C for 90 min under continuous agitation. Subsequently, 40 μL of N-methyl-N-(trimethylsilyl)trifluoroacetamide (MSTFA) was added, and the reaction mixture was further incubated at 37 °C for 30 min with agitation to complete trimethylsilylation. Immediately after derivatization, the samples were analyzed by GC–MS. The extracts were analyzed with an Agilent 7890A gas chromatograph coupled to a Pegasus 4D GCxGC-TOFMS mass spectrometer (LECO, St. Joseph, MI, USA). The compounds were separated using a DB-5T bonded-phase fused-silica capillary column (30 m length, 0.25 mm inner diameter, 0.25 μm film thickness) (J&W Scientific Co., USA). The GC oven temperature program was as follows: 2 min at 70 °C, raised by 10 °C/min to 300 °C and held for 10 min at 300 °C. The GC analysis lasted a total of 36 min. Helium was used as the carrier gas at a flow rate of 1 mL/min. One microliter of each sample was injected in splitless mode. The initial injector temperature was 40 °C for 0.1 min, after which the temperature was raised by 6 °C/min to 350 °C. The septum purge flow rate was 3 mL/min and the purge was turned on after 60 s. The transfer line and ion source temperatures were set to 250 °C. In-source fragmentation was performed at 70 eV. Mass spectra were recorded in the range 35–850 m/z. Data acquisition, automatic peak detection, mass spectrum deconvolution, retention index calculation and library searches were performed using LECO ChromaTOF software. To eliminate retention time (Rt) shift and determine retention indexes (RI) for each compound, an alkane series mixture (C-10 to C-36) was injected into the GC/MS system. The metabolites were identified automatically with a library search (NIST library); an analyte was considered identified when it passed a quality threshold, i.e., a similarity index (SI) above 700 and a matching retention index ±10. Artifacts (alkanes, column bleed, plasticizers, MSTFA and reagents) were identified using the same method, and then excluded from further analyses. To obtain accurate peak areas for the deconvoluted components, unique quantification masses for each component were specified and the samples were reprocessed. The untransformed raw data related to metabolome analysis have been deposited in the open Zenodo repository (doi 10.5281/zenodo.19570945). The final data tables were transferred into the MetaboAnalyst 6.0 platform (https://www.metaboanalyst.ca/) for statistical analysis.

### Statistics

The data tables used for the metabolome study were analyzed using the MetaboAnalyst 5.0 and 6.0 platform. The obtained data were normalized using the TIC approach. The ion intensities were transformed to log values, auto-scaled and all samples were grouped using categorical annotation and filtered against blanks (for untransformed GC–MS/MS data please see: File S2 available as Supplementary Data at *Tree Physiology* Online). The obtained data tables were used for statistical analysis. Principal component analysis (PCA), an unsupervised pattern recognition method, was performed to examine variation in the root metabolomes and illustrate the relationships among the samples. To validate the statistical significance of the identified differential metabolites, independent comparisons were made: all four analyzed variants (two provenances in two seasons) were compared using multi-sample ANOVA (α = 0.05). Only metabolites that had significantly different abundances (according to ANOVA) were presented on the heat map. Finally, Student’s *t*-tests were performed to compare spring and autumn metabolome for particular provenance, as well as to compare KO and PPN metabolome for particular season. Differences were considered significant at *P* ≤ 0.05 (False Discovery Rate (FDR) ≤ 0.05), and only metabolites meeting both the significance threshold and a fold-change criterion (FC ≥ 2.0 for increased abundance or FC ≤ 0.5 for decreased abundance) are presented in the main manuscript.

The non-equal variances tests were performed, where necessary. Additionally, quantitative enrichment analyses based on the KEGG library were performed for seasons comparisons.

Biometrical and biochemical results were subject to ANOVA followed by t test (spring vs autumn comparison) and Tukey’s HSD post hoc test (comparison across all variants). Differences were considered significant at *P* ≤ 0.05. These analyses were performed using the JMP Pro 13.0.0 software (SAS Institute Inc.).

## Results

### Root metabolome

With use of untargeted GC–MS/MS analysis of root metabolome 160 compounds (162 hits) have been successfully identified in Scots pine fine roots (File S2 available as Supplementary Data at *Tree Physiology* Online). This study focused on fine roots, including the fraction of mycorrhizal root tips, in which the detected compounds may be of both plant and fungal origin. Fungal biomass was not directly quantified in this study; however, based on recent literature on *P. sylvestris* and ectomycorrhizal systems, it is estimated to account for less than 5–10% of the total mass of the analyzed roots (Satomura et al. 2003, Wallander et al. 2005). We detected numerous compounds that varied in abundance between the analyzed provenances, but the abundance of many of those compounds was only slightly shifted (did not reach the FC > 2; File S2 available as Supplementary Data at *Tree Physiology* Online).

The PCA showed that the first two principal components jointly explained 52.4% of the total variance in the Scots pine root metabolomes (Figure 2). The PCA score plot showed that PPN spring root metabolome is distinguishable from the remaining variants. Thus, the root metabolome differed significantly between the analyzed provenances (KO vs PPN) in spring, whereas in autumn fine roots of both provenances were characterized with relatively similar metabolite abundances (Figure 2).

**Figure 2:**
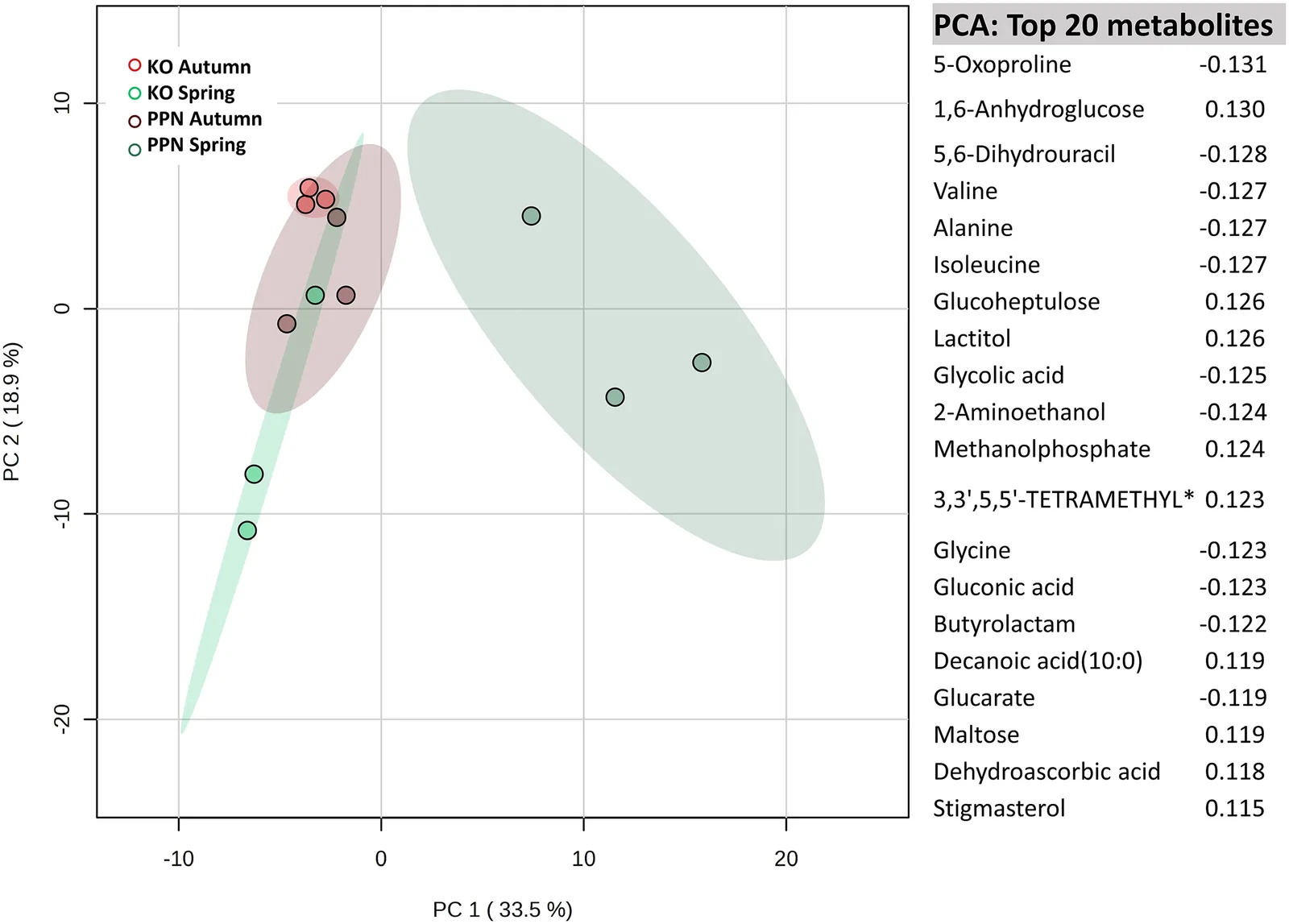
Principal component analysis of metabolite composition detected in fine root extracts (*n* = 3). PC1, the first principal component; PC2, the second principal component. On the right: top 20 metabolites contributing to PC1 (with loading1 values). ^*^ 3,3′,5,5’-TETRAMETHYL-4,4’-DIMETHOXY-1,1’-BIPHENYL.

Among the metabolites having the greatest impact on PCA distribution, nitrogen compounds predominated, primarily amino acids (Figure 2). In the KO, the differences between spring and autumn root metabolome were smaller (according to the PCA analysis; Figure 2), and the number of compounds differing in abundance between spring and autumn fine roots metabolomes, was lower, compared with PPN (Table 1).

**Table 1:** Compounds differ in their abundance between particular two pair comparisons (*t*-tests).

	**FC**	**log2(FC)**	***P*-value (adjusted)**	**−log(10)*P***
**Spring (PPN vs KO)**				
Decanoic acid(10:0)	43.596	5.4461	0.0075241	2.1235
Gamma-linolenic acid	7.318	2.8714	0.046692	1.3308
(1R,2R)-1-(5-HYDROXY-^a^	4.6091	2.2045	0.03658	1.4368
FLEXIBILENE	3.6263	1.8585	0.046692	1.3308
(+−)-Alpha-tocopherol	3.0049	1.5873	0.036874	1.4333
Aspartate minor	0.13167	−2.925	0.03658	1.4368
Pyrophosphoric acid	0.082808	−3.5941	0.0046374	2.3337
Glycolic acid	0.046658	−4.4217	0.0063525	2.1971
L-Valine	0.042326	−4.5623	0.046692	1.3308
L-Isoleucine	0.029168	−5.0995	0.0083147	2.0802
Glycine	0.015528	−6.009	0.017355	1.7606
L-Alanine	0.009749	−6.6805	0.0022886	2.6404
L-5-Oxoproline	0.009419	−6.7301	0.046692	1.3308
4-Aminobutyric acid	0.004205	−7.8937	0.03658	1.4368
2-Aminoethanol	0.001876	−9.0585	0.0039773	2.4004
**Autumn (PPN vs KO)**				
Beta-D-(+)-glucose	0.10547	−3.2451	0.0024484	2.6111
Aspartate minor	0.18727	−2.4168	0.018835	1.725
Lactobionic acid	0.34665	−1.5284	0.0039861	2.3995
L-Glutamic acid	0.35956	−1.4757	0.032372	1.4898
Glucarate	0.43716	−1.1938	0.024349	1.6135
Alpha-lactose	0.43828	−1.1901	0.0039861	2.3995
Galactinol	0.44817	−1.1579	0.024349	1.6135
**KO (spring vs autumn)**				
Indole-3-acetaldehyde	13.319	3.7354	0.012475	1.904
Lactulose^b^	0.46746	−1.0971	0.0054781	2.2614
D-Panose	0.39527	−1.3391	0.049799	1.3028
Glucarate	0.3665	−1.4481	0.040296	1.3947
Raffinose	0.32666	−1.6141	0.045176	1.3451
Inulotriose	0.3266	−1.6144	0.045176	1.3451
Aspartate minor	0.2754	−1.8604	0.049799	1.3028
L-Asparagine	0.2501	−1.9994	0.045176	1.3451
lactobionic acid	0.22207	−2.1709	0.013095	1.8829
Alpha-lactose^b^	0.10265	−3.2842	0.0054781	2.2614
D-(+)-Raffinose	0.012192	−6.3579	0.012475	1.904
**PPN (spring vs autumn)**				
Decanoic acid(10:0)	16.505	4.0449	0.034122	1.467
Beta-D-(+)-glucose	11.906	3.5736	0.0025774	2.5888
D-(+)-trehalose	3.4958	1.8056	0.034122	1.467
Methanolphosphate	2.9166	1.5443	0.027989	1.553
Glucose non-meox	2.6629	1.413	0.009481	2.0231
Alpha-lactose^b^	0.30551	−1.7107	0.034122	1.467
L-(−)-Malic acid	0.27355	−1.8701	0.034122	1.467
Urea	0.24065	−2.055	0.027989	1.553
Aspartate minor	0.19364	−2.3686	0.034122	1.467
Diethanolamine	0.093337	−3.4214	0.027989	1.553
L-Isoleucine	0.046743	−4.4191	0.034122	1.467
Butyrolactam	0.026776	−5.2229	0.034122	1.467
Glycolic acid	0.025976	−5.2667	0.0025774	2.5888
Glycine	0.018786	−5.7342	0.034122	1.467
L-Alanine	0.014375	−6.1203	0.027989	1.553
L-5-Oxoproline	0.009001	−6.7956	0.034122	1.467
2-Aminoethanol	0.001973	−8.9856	0.034122	1.467

Metabolites showing larger changes in abundance (FDR < 0.05; fold-change cutoff FC ≥ 2.0, corresponding to FC ≥ 2.0 for upregulated and FC ≤ 0.5 for downregulated metabolites) are presented in this table. For a full list of metabolites that meet the *t*-test criteria (α = 0.05), see File S2 available as Supplementary Data at *Tree Physiology* Online.

^a^(1R, 2R)-1-(5-HYDROXY-3-HYDROXYMETHYL-3-PENTENYL)-2,4A-DIHYDROXYMETHYL-1,2,3,4,4A,7,8,8A-OCTAHYDRONAPHTHALENE-5-CARBOXYLIC ACID4A,5-LACTONE.

^b^Compounds not commonly reported in roots or soil metabolomes and may reflect analytical ambiguity among structurally similar disaccharides and will be not further discussed.

Hierarchical analysis of compounds that differed significantly in their abundance (according to ANOVA; Table 1) reflected both the seasonal differences and the effects of Scots pine seed origin locations (see classes on heatmap; Figure 3). According to the hierarchical analysis, the distance between spring and autumn was bigger for PPN than for KO, with spring PPN metabolome differing from all remaining variants, whereas both autumn metabolomes (KO and PPN) were relatively similar to each other (Figure 3). This result is consistent with the PCA map (Figure 2).

**Figure 3:**
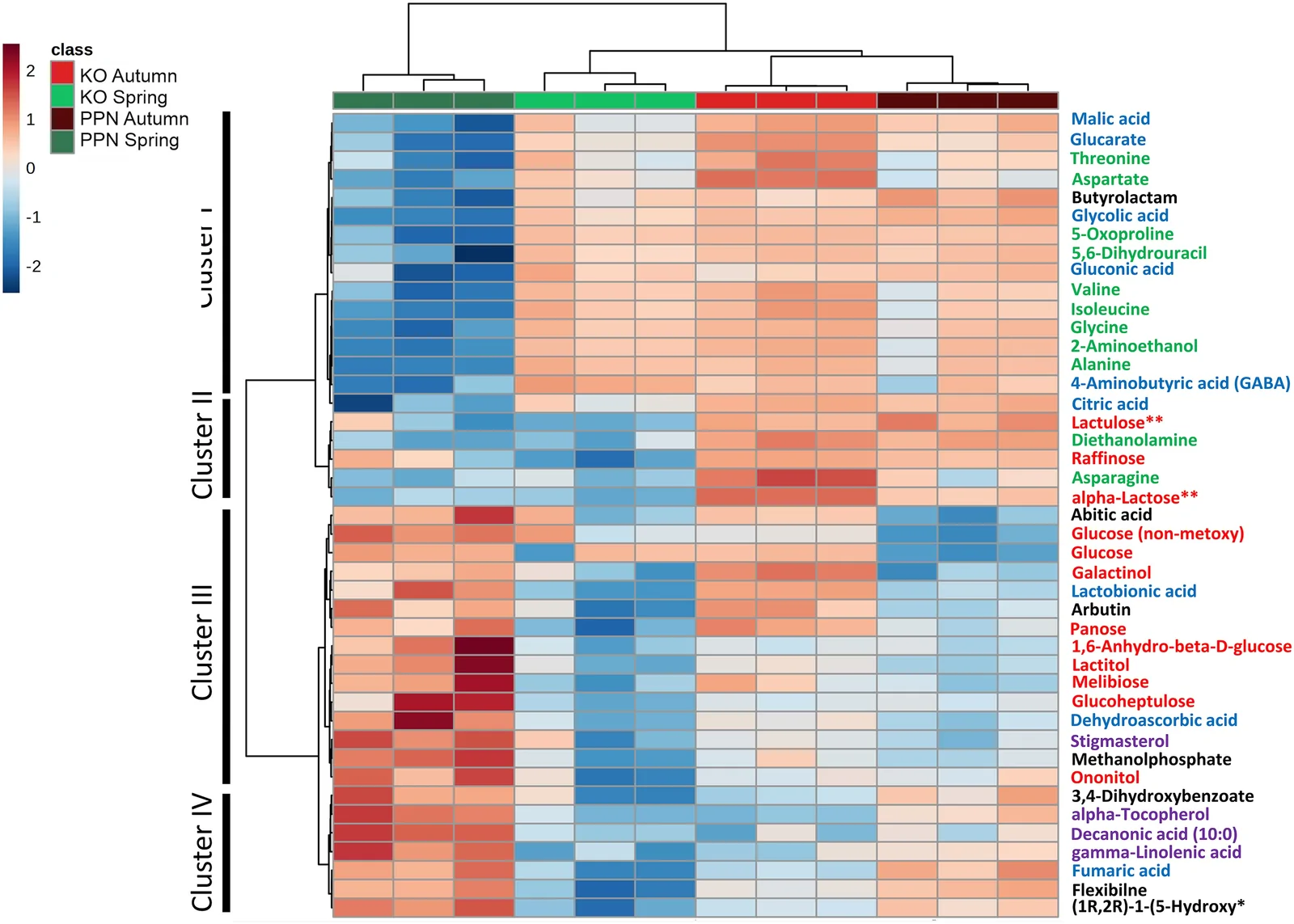
Heat map analysis combined with hierarchical cluster analysis showing determined metabolites that were differentially abundant (*P* < 0.05, ANOVA, without FC cut-off) between the analyzed variants (fine root metabolomes of KO and PPN, both in spring and autumn). Intensity values were log2-transformed, batch-corrected and Z-scored rowwise. Heatmap: blue boxes, minimal abundance; red, maximal abundance. Compound names color code: blue—organic acids; green—amino acids and other main N-compounds; red—carbohydrates and their alcohols; purple—lipidic compounds; black—remaining compounds. For clusters (I–IV) explanation, please see the main text. ^*^(1R, 2R)-1-(5-HYDROXY-3-HYDROXYMETHYL-3-PENTENYL)-2,4A-DIHYDROXYMETHYL-1,2,3,4,4A,7,8,8A-OCTAHYDRONAPHTHALENE-5-CARBOXYLIC ACID4A,5-LACTONE. ^**^Lactose and its derivatives are compounds that are not commonly reported in roots or soil metabolomes and may reflect analytical ambiguity among structurally similar disaccharides and will be not further discussed.

According to the heat map analysis, in spring the fine roots’ metabolome of trees originating from the cooler and wetter location (PPN) was characterized with the lowest abundance of majority of N-compounds, including numerous amino acids, compared with all remaining metabolomes (cluster I in Figure 3). Moreover, among differently-abundant N-compounds detected in our study, all were enriched in autumn, compared with spring, for both PPN and KO (Table 1). Lipidic compounds were detected as more abundant in PPN roots in spring when compared with KO spring and PPN autumn roots (Figure 3 cluster IV and Table 1). Among those compounds, we detected saturated decanoic acid, characterized with one of the highest FCs, however, unsaturated lipidic acids or sterols were also enriched in PPN spring roots (Figure 3 clusters II and III, Table 1 and File S3 available as Supplementary Data at *Tree Physiology* Online). The differences in carbohydrates were more variable (Figure 3, Table 1). Several main carbohydrates, like glucose or trehalose were more abundant in PPN roots in spring compared with autumn, without a similar adjustment in KO roots. In contrast, raffinose or panose were more abundant in autumn than in spring in roots of KO provenance, but not in PPN. Organic acids adjustments varied between analyzed variants and most often were less intense, not crossing the FC ≥ 2 (Files S2 and S3 available as Supplementary Data at *Tree Physiology* Online), except for glycolic and malic acids (Table 1). Tocopherol and dehydroascorbic acid, compounds commonly linked with the anti-stress response, were most abundant in the PPN spring roots (Figure 3, Table 1).

In roots of the local provenance, the most enriched metabolic pathway was related to butanoate metabolism, followed by numerous pathways related to AAs metabolism (Figure 4a), reflecting numerous N-compounds enriched in KO autumn roots (Figure 3, Table 1). In the autumn KO roots we also detected increased levels of glucarate (Figure 3).

**Figure 4:**
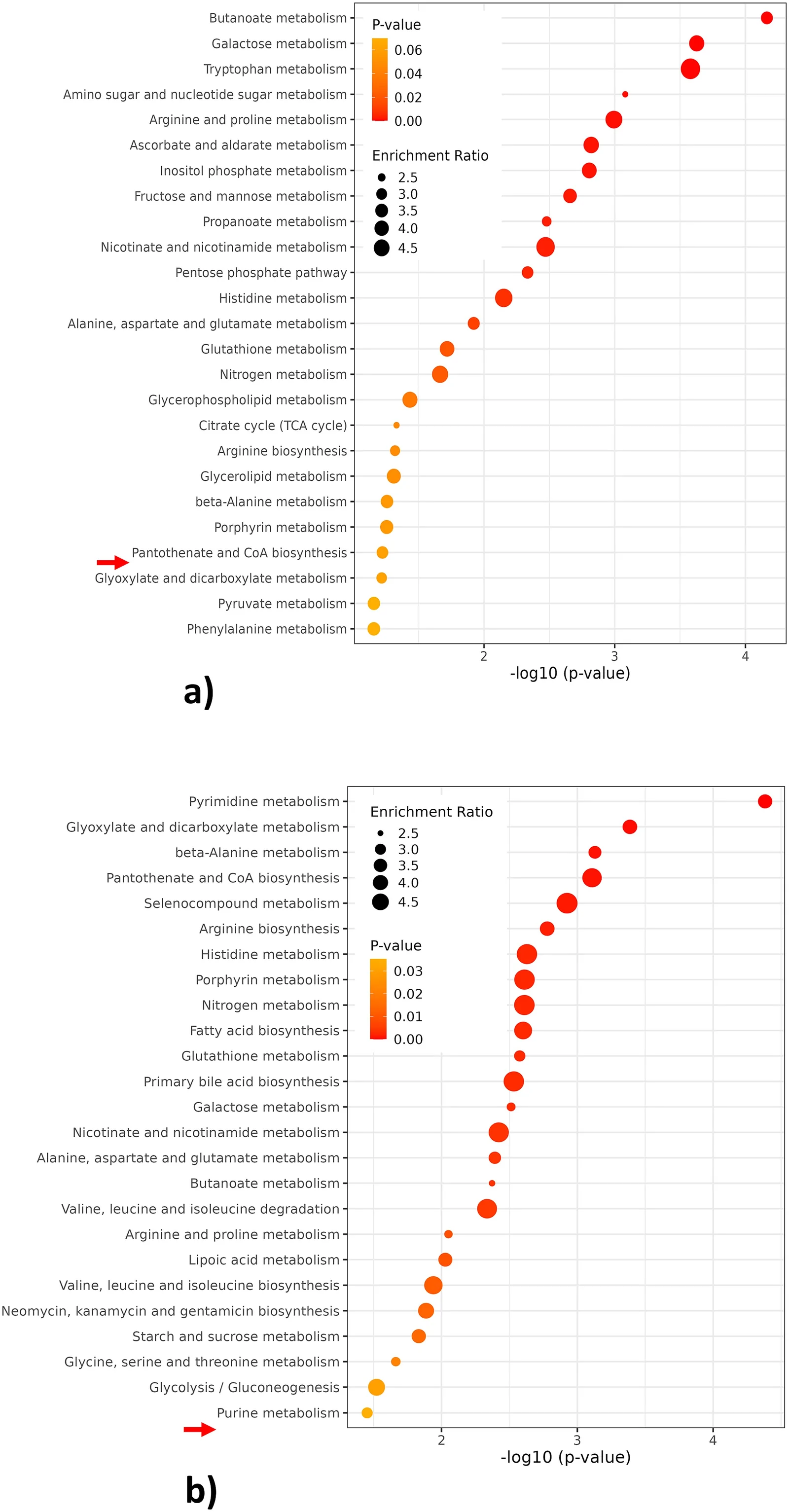
Pathway enrichment. Graphs present the results of the quantitative pathway enrichment analyses expressed differences between spring and autumn fine root metabolome for KO (a) and PPN (b). Analysis was performed on the basis of the KEGG library using the MetaboAnalyst 6.0 (https://www.metaboanalyst.ca/) platform. Compounds and their abundances were subjected to the analysis. The metabolic pathways are represented according to their scores from the enrichment (vertical axis; (−log(*P*)) and topology analyses (pathway impact, horizontal axis). Arrow: cut off (fulfilling the criterion *P* < 0.05 and False Discovery Rate = 0.05).

The most enriched in PPN roots were pathways related to pyrimidine, and glyoxylate (dicarboxylate) metabolism. Among the significantly enriched pathways, the signals of alteration of lipid metabolism were detected (fatty acid or lipoic acid metabolism; Figure 4b). Nevertheless, the most numerous enriched pathways were related, similarly as for KO, to amino acids metabolism (Figure 4b).

### Root biochemistry

For majority of analyzed traits the effect of season was significant, in contrast to provenance effect being significant only for SC (%) (Table 2). In fine roots of *P. sylvestris* collected in spring, the percentage of total nitrogen was lower than in autumn for both analyzed provenances (KO and PPN), but significant seasonal differences were detected only for PPN trees (Figure 5a). Total carbon level in fine roots did not differ between provenances, but it was significantly higher in autumn, compared with spring (Figure 5b). Interestingly, the higher total levels of carbon detected in autumn did not correspond with NSC levels. Both soluble NSCs (Figure 5c) and starch (Figure 5d) concentrations were significantly higher for both provenances in spring compared with autumn roots. Moreover, in accordance with the ANOVA result, indicating a significant effect of provenance (Table 2), the concentrations of both SC and starch tended to be higher in PPN roots compared with KO (Figure 5c and d). The total protein content, estimated based on protein extraction yield, did not differ among the analyzed variants (Figure 5e), nor did the total phenolic content (Figure 5f). Higher levels of reactive oxygen species, estimated based on H₂O₂ concentration, were detected in roots collected in autumn, irrespective of provenance (Figure 5g). This increase, however, was not fully paralleled by changes in lipid peroxidation. Malondialdehyde, a marker of cellular membrane damage (Shah et al. 2001), remained low and stable across seasons in KO roots. By contrast, PPN roots displayed higher MDA levels overall, with a significant seasonal increase observed in autumn (Figure 5h). Proline, partially correlated with changes in MDA, but was significantly higher (compared with the other metabolomes) only in the autumn roots of PPN (Figure 5i).

**Table 2:** ANOVA table for structural and biochemical root traits for differences among origin and season.

	**Root traits**	**Source of variation**	**d.f.**	** t ratio**	*** P***
Structural root traits	Specific root area (SRA)	Provenance (P)	1	0.83	0.412
		Season (S)	1	**5.79**	**<0.0001**
		P × S	3	0.15	0.880
	Root tissue density (RTD)	Provenance (P)	1	0.98	0.330
		Season (S)	1	**5.85**	**<0.0001**
		P × S	3	0.13	0.896
	Root branching ration (RBR_1,2_)	Provenance (P)	1	**−2.57**	**0.013**
		Season (S)	1	−1.08	0.286
		P × S	3	0.79	0.433
	Length of root with diameter class (0–0.5 mm)	Provenance (P)	1	1.03	0.307
		Season (S)	1	**3.79**	**0.0004**
		P × S	3	0.94	0.351
	Length of root with diameter class (0.5–1 mm)	Provenance (P)	1	0.33	0.745
		Season (S)	1	**−3.70**	**0.0005**
		P × S	3	−0.58	0.566
Biochemical root traits	Carbon concentration (%)	Provenance (P)	1	−0.62	0.537
		Season (S)	1	4.61	0.537
		P × S	3	**0.21**	**<0.0001**
	Nitrogen concentration (%)	Provenance (P)	1	−1.45	0.154
		Season (S)	1	**3.62**	**0.0007**
		P × S	3	−0.79	0.433
	Soluble carbohydrates concentration (%)	Provenance (P)	1	**−2.16**	**0.035**
		Season (S)	1	**−11.63**	**<0.0001**
		P × S	3	1.25	0.217
	Starch concentration (%)	Provenance (P)	1	−1.68	0.098
		Season (S)	1	**−10.41**	**<0.0001**
		P × S	3	1.67	0.100

Bold values indicate statistically significant effects (*P* < 0.05).

**Figure 5:**
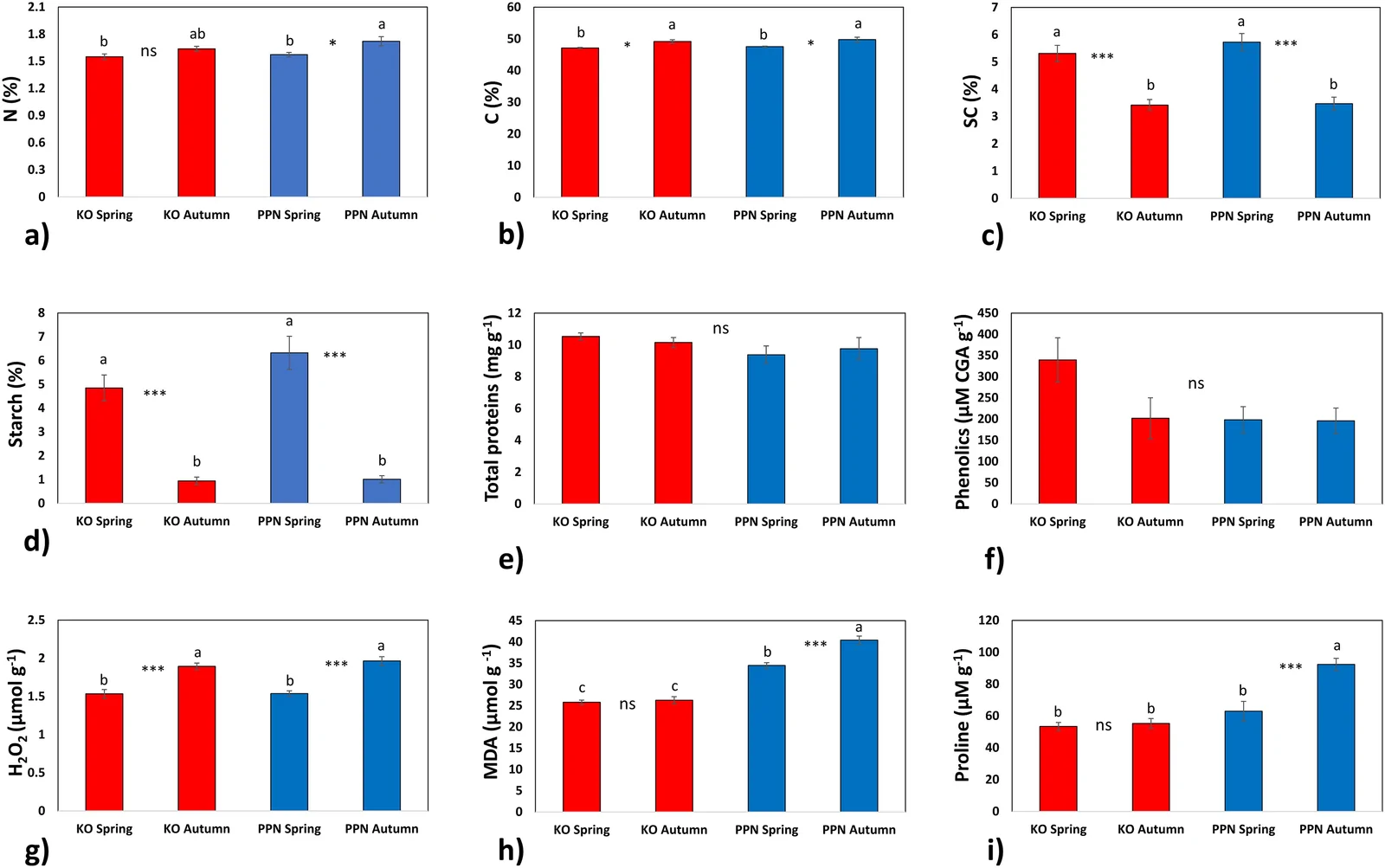
Root biochemistry. Nitrogen (a) and carbon (b) percentage in analyzed roots (*n* = 3). Soluble carbohydrates (c) and starch (d) percentage in analyzed roots (*n* = 6). Estimated total protein concentrations (e; *n* = 6). Total phenolic compounds concentrations (normalized against chlorogenic acid (CGA) as a standard (*n* = 5) (f). Hydrogen peroxide (H_2_O_2_) (g) and malondialdehyde (MDA) concentrations (*n* = 6) (h). Proline concentrations (*n* = 6) (i). Values are expressed as the mean ± SD. Different letters represent significant differences between particular variants according to HSD post hoc test. Asterisks: Differences spring and autumn for particular variant (^*^P < 0.05; ^***^P < 0.0005); ns: not significant. (a–d and f) Features analyzed in dry root mass; (e and g–i) Features analyzed in fresh root mass.

The FTIR analysis of the main structural carbohydrates of the root cell walls, that is the intensities of all selected bands representing cellulose and hemicellulose as well as band characteristic of lignin, revealed that the abundances of analyzed structural carbohydrates were higher in the roots collected in autumn for both analyzed provenances (Figure 6a and b).

**Figure 6:**
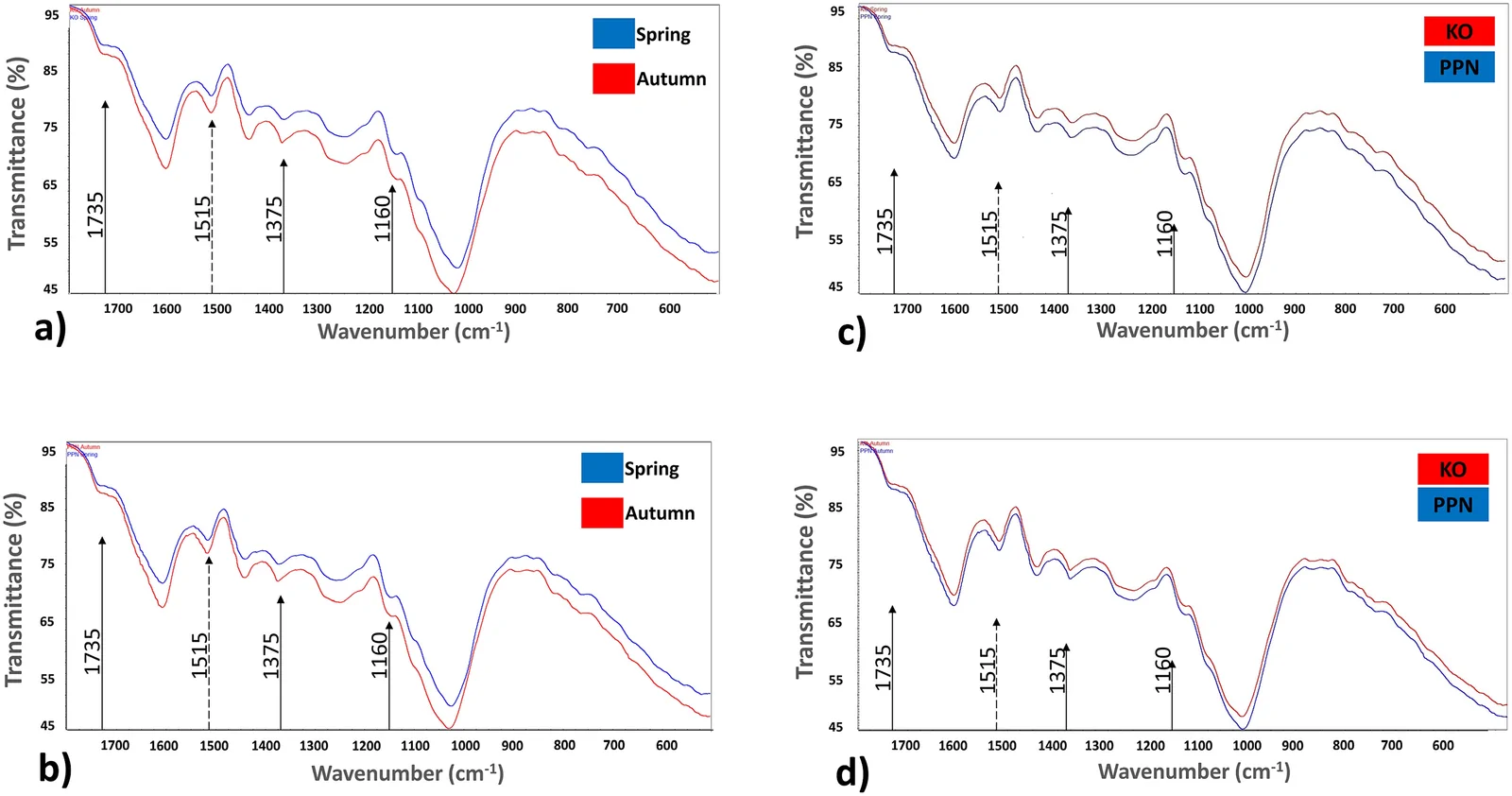
Spectra comparison between Scots pine roots collected in spring and autumn for KO (a) and PPN (b) provenance. FTIR spectra comparison between both proveniences (KO and PPN) presented for root samples collected in spring (c) and autumn (d). The most important wavenumbers has been marked with arrows with solid lines representing bands characteristic for cellulose and hemicellulose and dashed line representing band characteristic for lignin. The analysis was focused on the selected bands of captured spectra, namely 1515 cm^−1^ representing skeletal aromatic rings of lignin, 1375 cm^−1^ corresponding to the deformation of C–H cellulose and hemicelluloses, 1160 cm^−1^ (vibration of C–O–C in cellulose and hemicelluloses) and 1735 cm^−1^ associated with C-O stretching vibrations in unconjugated ketones, carboxyl groups and ester groups, predominantly found in hemicelluloses.

Our results revealed also differences in cell wall carbohydrates deposition between roots of analyzed Scots pine provenances within particular season. In both spring (Figure 6c) and autumn (Figure 6d) roots, the higher amounts of structural carbohydrates were detected in PPN, compared with KO roots. Moreover, in autumn, the differences between both provenances tended to be less significant (distance between both spectra was smaller: Figure 6c  vs  Figure  6d).

### Root structure

The season (spring vs autumn) significantly influenced the analyzed structural root traits (Table 2, Figure 7) Both, specific root area (SRA; Figure 7a) and RTD (Figure 7b) were significantly lower in roots collected in spring than in autumn. We also detected differences in morphology of root fractions.

**Figure 7:**
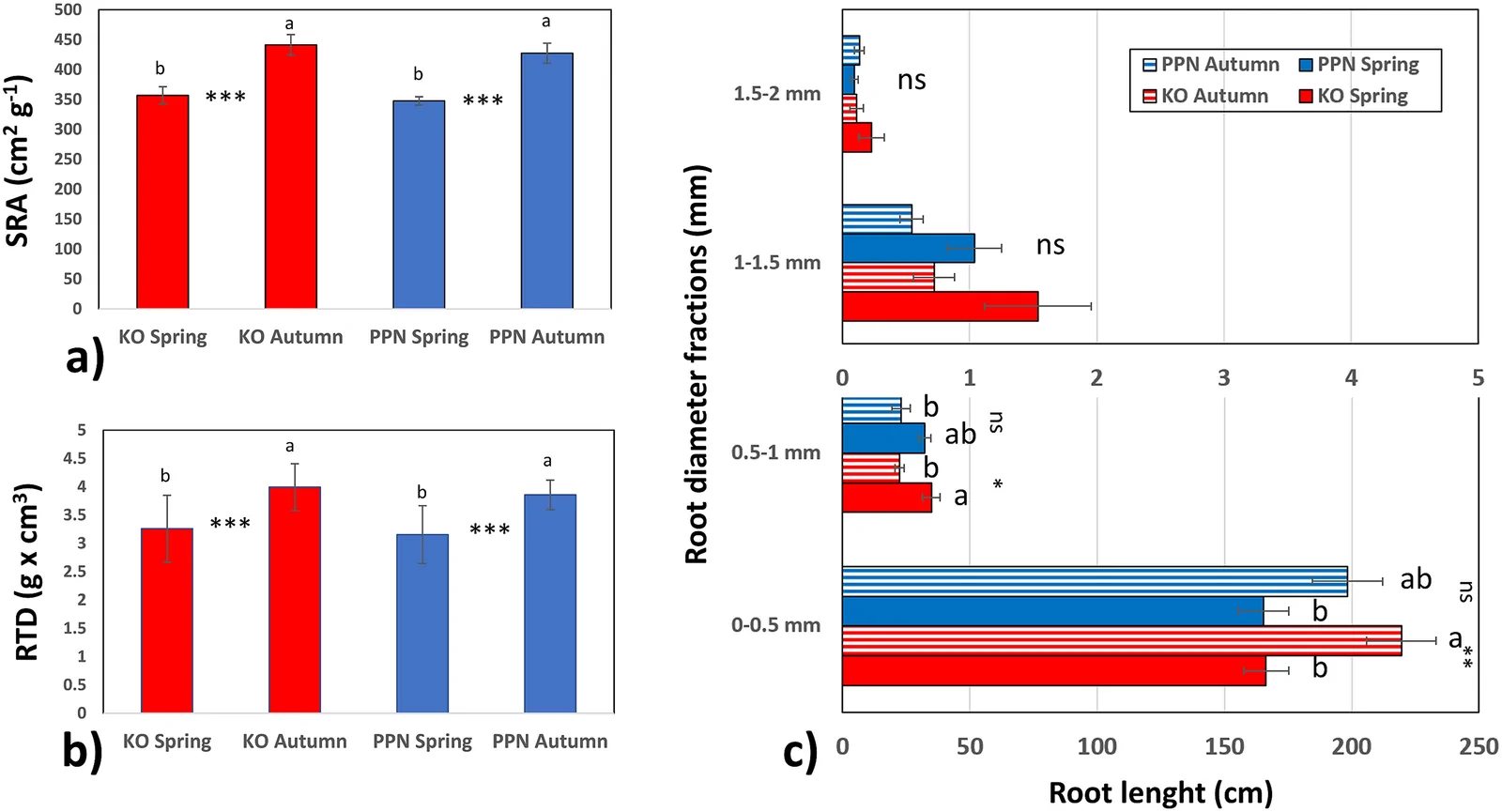
Root sample structure. A representative fine root sample (harvested in spring and autumn and corresponding to the root samples used for GC–MS/MS experiment). Specific root area (SRA) (a), root tissue density (RTD) (b) and root diameter fractions (c). Values are expressed as the mean (*n* ≥ 10) ± SD). Different letters represents significant differences between particular variants according to HSD post hoc test. Asterisks: differences between spring and autumn for particular variant (^*^P < 0.05; ^**^P < 0.005; ^***^P < 0.0005); ns: not significant.

The thinnest roots—the major root fraction with diameter ranging between 0 and 0.5 mm—were more abundant (their summarized length was higher) in autumn compared with spring for both provenances. In contrast, roots with diameter between 0.5 and 1 mm showed the opposite trend, and were more frequent in samples collected in spring than in autumn (significantly for KO roots; Figure 7c). Among the examined structural traits, provenance had a significant impact only on root branching ratio (RBR_1,2_; Table 2).

## Discussion

Metabolite profiles may be highly heritable in plants, indicating strong genetic control over metabolic phenotypes (e.g., Guo et al. 2020, Gemmer et al. 2021). At the same time, the metabolome is considered to be the OMIC level that responds most intensively to environmental changes (e.g., Cao et al. 2019, Sobuj et al. 2021). This combination makes metabolomics especially useful for examining long-term provenance effects in mature trees grown in common-garden settings (Hunziker et al. 2024). However, since the samples were collected under uncontrolled, natural field conditions (Poorter et al. 2012) and only two seasonal time points were studied, our findings should be seen as indications of provenance-related differences in metabolomics rather than direct proof of particular physiological processes. Furthermore, because mother-tree identity was not preserved in the historical trial and metabolomic replicates were pooled across trees, our results capture provenance-level patterns rather than family-level genetic variation.

Small-scale heterogeneity in soil properties can influence root physiological traits even within otherwise uniform forest stands. However, in our study, all trees were grown for more than 50 years in the same long-term common garden, and the sampling integrated root material from multiple individuals across the plot. This minimizes the likelihood that observed metabolomic differences are driven by microsite edaphic variation (Szuba and Lorenc-Plucińska 2015, Bragazzi et al. 2026), instead of reflecting provenance- and season-related effects. Our pooling strategy also partially addresses the next limitation of this study—the relatively low sample size (use of only three biological replicates) of GC–MS/MS extracts per experimental variant. Each replicate represented material collected from multiple trees, thereby reducing the influence of individual-tree variability and increasing the representativeness of each sample (Takele Assefa et al. 2020, Bragazzi et al. 2026). Nevertheless, further broader-scale studies comparing the metabolomes of adult trees from different provenances are warranted.

Long-term exposure to the shared environmental conditions of the Experimental Forest ‘Zwierzyniec’ resulted in only a small number of significantly altered metabolites among trees of different origins, suggesting a high degree of homeostasis in the analyzed trees (Hunziker et al. 2024). Our study found, however, significant seasonal changes in the fine root metabolome of Scots pine trees transplanted from colder, wetter conditions compared with those from the local area. The metabolites that most clearly distinguished the provenances included nitrogen-containing compounds, soluble carbohydrates and lipids, confirming our initial hypothesis that the root metabolomes of the two provenances would stay distinguishable even after over 50 years of growth under identical environmental conditions.

Our results also support previous research showing that seasonal variations may influence elemental stoichiometry—which is closely tied to plant metabolomic profiles (Rivas-Ubach et al. 2012)—as well as the abundance of various plant compounds (Renaut et al. 2005).

In addition to these differences in metabolite profiles, we also found variations in soluble carbohydrate content across different provenances, as well as differences in fine root branching ratios. These patterns, along with increased levels of MDA and proline, may suggest that the transplanted provenance may not have fully adjusted to the warmer, drier conditions in Kórnik, leading to stress responses to drought and high temperatures (Isaac-Renton et al. 2018, Chauvin et al. 2019, Ghosh et al. 2022). These findings confirm that both provenance and season interact to shape root physiology, reflecting a combination of genetic legacy and acclimatory responses. Importantly, the present experimental design does not allow these components to be separated quantitatively. Our study showed that long-term provenance effects influence multiple interconnected metabolic networks rather than just individual metabolites. We confirmed the hypothesis that differences in metabolomics related to provenance involve pathways linked to carbon and nitrogen metabolism, membrane lipids and stress compounds. An interesting question is whether trees from colder and wetter environments keep metabolic traits that help them adapt to cold conditions. We found some evidence to support this idea. The roots of trees from the PPN region had higher levels of cell wall carbohydrates and starch and exhibited signs of altered lipid metabolism in the spring. These alterations are often linked to cold tolerance and structural strength (Munné-Bosch 2005, Aboobucker and Suza 2019).

The PPN roots in spring showed increased levels of decanoic acid, sterols and linolenic acid, indicating membrane remodeling, tied to cold acclimation (Iivonen et al. 2004, Cheong et al. 2022, Jahed et al. 2023). Cold stress is also linked to higher saturation of free fatty acids (Iivonen et al. 2004), and such increases have been seen in Scots pine roots at the start of the growing season (Jahed et al. 2023). In line with this, we found decanoic acid, the most enriched lipid, in PPN spring roots, along with higher levels of membrane-modulating lipids and sterols (Aboobucker and Suza 2019). At the same time, lipids are altered by diverse stressors. While drought typically reduces fatty acid unsaturation (Yin et al. 2024), the opposite trend observed here may reflect inherited cold-adaptation pathways rather than drought responses. However, lipid shifts may also be mediated by ectomycorrhizal associations, as sterols and fatty acids are crucial for plant–fungal communication under stress (Sebastiana et al. 2014). Consequently, our study does not allow for a definitive distinction between these physiological mechanisms or for determining whether these metabolites are solely markers of inherited cold adaptation. Nevertheless, the observed changes may represent components of broader protective responses that contribute to the maintenance of membrane integrity under diverse environmental stresses. Our results showed that provenance-related differences were greatest in the spring. This observation coincides with the spring deacclimation period, during which Scots pine undergoes extensive physiological and metabolic reorganization (Saranpää and Nyberg 1987, Iivonen et al. 2004).

While the spring metabolome of PPN roots was most distinct from other ones in our study, the autumn metabolomes of both provenances became notably more similar. This suggests that seasonal physiological processes can partly override provenance-specific metabolic signatures later in the growing season. Since only two seasonal sampling points were analyzed, distinguishing among these mechanisms is challenging. However, the continued influence of provenance effects after over 50 years of identical environmental growth indicates that genetic background still shapes root metabolism in mature trees. This was supported by the PCA, and hierarchical clustering clearly separated the spring metabolomes of PPN from all other provenance and season combinations, while autumn profiles showed more similarities between provenances. Changes in abiotic conditions, such as temperature, moisture and CO₂ levels, during different seasons drive metabolic adjustments and influence the expression of genotype-specific traits (Ji et al. 2022, Maitra et al. 2024*b*). A similar pattern was observed in our study, as seen in a common garden study on *Prunus spinosa* by Wanjiku and Bohne (2021), where differences in soluble sugars, starch and macroelements among genotypes peaked in the spring but decreased by summer and autumn. These variations coincided with phenological shifts related to geographic origin. In our study in spring, PPN roots accumulated osmoprotective sugars such as trehalose, as well as stress-associated lipids, which may indicate metabolic adjustments to environmental mismatch (Szuba et al. 2022, Wu et al. 2022).

In the autumn, aboveground growth typically stops as air temperatures drop (Heide and Prestrud 2005), while buffered soil temperatures allow roots to remain metabolically active until temperatures fall below 0 °C (Mao et al. 2013). Thus, although we did not investigate root growth dynamics, all roots analyzed in our study were likely still active in the autumn, as is also indicated by the increased H₂O₂ level, detected for both provenances, which here we linked rather with a higher physiological activity, than with a stress response (Mucha et al. 2019). Prolonged root activity may be significant for genotypes from colder climates, as suggested by studies along arctic elevation gradients (Blume-Werry et al. 2016). This may explain why differences between PPN and KO were less pronounced in the autumn than in the spring.

One of the most notable trends in Scots pine roots was the increased abundance of low-molecular-weight nitrogen-containing metabolites metabolites in the autumn, which was supported by a rise in nitrogen content. The fact that PPN trees had higher root nitrogen content in autumn roots may support the idea that they may have better nutrient acquisition, storage or nitrogen resorption. This is beneficial for trees with short growing seasons (Reich et al. 2014, You et al. 2023). Our findings suggest that prolonged exposure to a shared environment did not completely eliminate provenance-related metabolic differences (Beuker et al. 1998, Oleksyn et al. 2001). Nitrogen-containing compounds are known to help plants cope with stress. When trees are exposed to high temperatures and drought, they typically increase the production of osmoprotectants like sucrose and amino acids such as proline, one of the most common free amino acids and a multifunctional low-molecular-weight antioxidant and osmoprotectant in plants, glycine and asparagine (Ghosh et al. 2022, Hunziker et al. 2024). These substances were also found in drought-stressed *Pinus taeda* seedlings (Wu et al. 2022). It cannot be ruled out that elevated MDA or proline levels, detected in PPN roots, may indicate an increased stress response in those trees (Szuba et al. 2022).

Our results suggest that these seasonal shifts mainly involved soluble nitrogen pools rather than enhanced protein synthesis, as the lack of corresponding increases in protein levels was observed. We propose that nitrogen remobilization and storage could explain the observed metabolomic changes in autumn, although other physiological factors might also play a role. This seasonal nitrogen redistribution, common in perennial trees, is typically associated with seasonal nitrogen remobilization, resorption and storage before winter dormancy, rather than continued biomass growth (Cooke and Weih 2005, Millard and Grelet 2010, Babst and Coleman 2018). It is known that the dynamics of nitrogen in roots are also closely linked to interactions with soil microbiota, particularly ectomycorrhizal fungi, essential partners of Scots pine, which enhance nitrogen availability through symbiotic exchange (Smith and Read 2008). Indeed, it has been shown that, within the genus *Pinus*, soil microbiota may influence the seasonal accumulation of low-molecular-weight nitrogen compounds such as amino acids in roots (Meier et al. 2017). However, the role of nitrogen remobilization and storage processes, interactions with rhizosphere organisms, and other physiological factors as potential causes of the changes in the root metabolome of Scots pine observed in our study requires further investigation.

Finally, our study indicates distinct seasonal changes in carbohydrate content, in line with previous research linking these patterns to genotype and environmental conditions (Meng et al. 2023). For instance, Rosinger et al. (2020) found low winter sugar levels in Scots pine roots, followed by a spring increase, likely due to carbohydrate transfer from storage organs to fine roots (Mei et al. 2015). In our data, we observed a spring increase in TNC, although changes in specific carbohydrates were limited. This may be due to Scots pine’s continued winter photosynthesis, which helps maintain carbohydrate reserves (Hansen and Beck 1994, Mao et al. 2013). Additionally, our GC–MS/MS method likely underrepresents oligosaccharides such as oligodextrins, which are common products of starch breakdown, due to technical limitations in detecting larger saccharides (Kailemia et al. 2014). We also detected elevated levels of trehalose in spring-PPN roots, an osmoprotective and storage sugar. Although trehalose may be present in plant cells and plays a role in carbon metabolism and various stress responses (Kleinschmidt et al. 1998, Szuba et al. 2022), it is also a compound commonly associated with fungi, and its increased abundance may indicate enhanced ectomycorrhizal associations (Kleinschmidt et al. 1998, Nehls et al. 2007). Nevertheless, our GC–MS/MS study does not allow us to determine the origin of this compound. Similar to the case of N-metabolites, without quantifying, e.g., microbial biomass, community composition or ectomycorrhizal colonization, we cannot attribute the observed metabolomic differences to microbial processes. Moreover, it was reported that root symbiosis may even suppress host metabolic pathways (e.g., glycolysis, TCA cycle) to divert carbon toward the fungal partner (Blaudez et al. 1998, Sebastiana et al. 2014). However, we observed no evidence of such shifts in our data, suggesting a similar carbon flux across all analyzed roots.

## Conclusions

Our study of mature *P. sylvestris* in a common garden suggests that the provenance leaves a persistent, seasonally modulated imprint on root function and metabolism, even decades after planting. The most noticeable differences appeared in early spring, when the trees from a colder area (PPN) had a distinct set of metabolites rich in lipids (including fatty acids and sterols) and low in nitrogen-containing compounds compared with the local provenance (KO). In both provenances, the metabolite profiles converged in autumn, with more amino acids and other nitrogen-containing compounds, and more structural carbohydrates in cell walls. The patterns, which are influenced by seasonal changes, potentially align with phenological adjustments linked to origin. Since many of the metabolites identified are involved in various abiotic stress-response pathways, and because microbial activity, nitrogen uptake and other physiological processes were not directly measured, the data cannot definitively identify the particular mechanisms behind the metabolomic patterns.

Nonetheless, these findings underscore the importance of considering seed origin in reforestation and afforestation programs, particularly as climate change reshapes growing conditions across Europe. Provenance-related traits may influence not only aboveground growth but also belowground resilience, nutrient cycling and symbiotic capacity. These results support the use of metabolomics as a sensitive tool for detecting long-term effects of provenance in forest trees.

## Supplementary Material

supplementary_materials_tpag126

## Data Availability

The main data generated or analyzed during this study are included in this published article and its supplementary information file. Additional datasets used and/or analyzed during this study are available from the corresponding authors upon reasonable request. The plant samples used in this study are maintained by the Institute of Dendrology Polish Academy of Sciences. The collection is housed at Parkowa 5, PL62035 Kórnik, Poland.
