# Supplementary material for: Provenance effect on the fine-root metabolome and structure of mature Scots pine revealed in a long-term common garden experiment
Source: Tree Physiol. 2026 Sep 8;46(10):tpag126. doi: 10.1093/treephys/tpag126 (PMC13652640; doi:10.1093/treephys/tpag126)
Supplement: Supplementary file 1 [file supplementary_materials_tpag126.zip › supplementary_materials_tpag126/Supplemenary_File_S3_Metabolome_data–volcano plots_Minor_R_clear_copy_tpag126.docx]

**Provenance effects on the fine-root metabolome and structure of mature Scots pine revealed in a long-term common garden experiment**

Agnieszka Szuba, Pulak Maitra, Katarzyna Hrynkiewicz, Izabela Ratajczak, Daniel J. Chmura and Joanna Mucha

**Supplementary File S3**

Metabolome data – volcano plots

Given that the study concerns provenance effects in mature trees that have been exposed to environmental pressures in the Zwierzyniec Experimental Forest for over half a century, we detected only modest changes in the metabolomes of the analyzed Scots pines originating from different provenances. Accordingly, we included in this Supplementary File compounds exhibiting less pronounced changes in abundance. However, it should be noted that these results (raw p < 0.05; FC cutoff = 1.1, corresponding to FC ≥ 1.1 for upregulated metabolites and FC ≤ 0.91 for downregulated metabolites) do not meet the cutoff criteria applied in the main manuscript and are not discussed in the main manuscript.

**Differences between provenances in particular season**

In the direct comparison betweenspring metabolomes of PPN and KO, the similar number of compounds was enriched in KO and PPN roots (Fig. S3.1a). However, the groups of those compounds were clearly different. Numerous N-compounds were more abundant in KO roots, whereas in PPN roots more abundant were mainly lipidic compounds and carbohydrates (Fig. S3.1a). The signals from central metabolism were not clear, as the malic acid was more abundant in KO spring roots, but fumaric acid and glucose-1-phosphate were enriched in spring PPN roots (Fig. S3.1a).

In autumn, majority of differently-abundant compounds had a higher concentrations in KO roots, but in contrast to spring, there is no clear dominance of particular metabolite class in KO or PPN roots. The more abundant carbohydrates, like glucose (a compound with the highest enrichment in this comparison), lactose, raffinose or panose were found in KO autumn roots, but also increases in aspartate or asparagine were found more abundant in the local, KO provenance (Fig. S3.1b). Among few compounds more abundant in PPN roots we found alpha tocopherol, but also oxoproline or fructose (Fig. S3.1b).

**Fig. S3.1** Differences between KO and PPN fine root metabolome presented for roots collected in spring (**a**) and autumn (**b**). Red points: compounds more abundant in KO, purple points: compounds more abundant in PPN fine roots. Names of selected compounds were presented on graphs. Results of raw t test (α=0.05), FC≥ 1.1 are presented on the graph.

**Spring vs autumn: seasonal differences for particular provenances**

The number of individual compounds differing significantly in terms of their seasonal abundance simultaneously for both provenances was limited, with only few characterized by the same direction of the seasonal changes, e.g. , alpha-lactose, aspartate, citric and malic acids, diethanolamine and glutarate, all being more abundant in autumn fine roots of both provenances (Fig. S3.2see also Supplementary File S2).

**Fig. S3.2** Differences between spring and autumn fine root metabolome presented for KO (**a**) and PPN (**b**). Red points: compounds more abundant in autumn, purple points: compounds more abundant in spring fine roots. Results of raw t test (α=0.05), FC≥ 1.1 are presented on the graph.

**KO roots**

In KO roots the vast majority of seasonally differently abundant compounds (according to t tests) were more abundant in autumn (Fig. S3.2a). The most enriched compounds in roots collected in late September (compared to the beginning of the growing season) were raffinose, alpha-lactose and products of its oxidation or isomerization: lactobionic acid and lactulose. The other more abundant in KO autumn roots compounds were amino acids, amines and its derivatives like L-asparagine, putrescine or diethanolamine. In autumn roots we detected also increased levels of glucarate, galacturonic acid (pectin building monomer; (Zdunek, Pieczywek, and Cybulska 2021)) and galactoinol (which is, similarly as raffinose, a defence signalling molecule (Meyer et al. 2018)) (Fig. S3.2a). Only four compounds were significantly enriched in spring roots of the local provenance (compared to autumn ones): indole-3-acetaldehyde (a precursor of IAA), allose, cysteinylglycine (aminothiol with antioxidant and redox control activity), and UDP-glucuronic acid (Fig. S3.2a and Supplementary File S2).

**PPN roots**

Similarly to KO, in autumn roots of trees originated from PPN the amino acids, amines and other N-compounds like urea were observed as more abundant than in spring, but the number of N-compounds enriched in autumn was much higher than in local, KO provenance. However, the glycolic acid was the most enriched compound in autumn PPN roots (compared to spring; Fig. S2b). Only the selected intermediates of glycolysis (glucose and 2,3-bisphosphglyceric acid: an isomer of 1,3-bisphosphoglycerate) were enriched in spring, whereas TCA cycle intermediates: citric and malic acid were more abundant in autumn PPN roots. In spring, more abundant were compounds related to membrane functioning: various lipid acids and sigmasterol (Fig. S3.2b and Supplementary File S2).
