# Supplementary material for: Provenance effect on the fine-root metabolome and structure of mature Scots pine revealed in a long-term common garden experiment
Source: Tree Physiol. 2026 Sep 8;46(10):tpag126. doi: 10.1093/treephys/tpag126 (PMC13652640; doi:10.1093/treephys/tpag126)
Supplement: Supplementary file 1 [file supplementary_materials_tpag126.zip › supplementary_materials_tpag126/Supplementary_File_S1_Common_garden_expanded_data_Minor_R_clear_copy_tpag126.docx]

**Provenance effect on the fine-root metabolome and structure of mature Scots pine revealed in a long-term common garden experiment**

Agnieszka Szuba, Pulak Maitra, Katarzyna Hrynkiewicz, Izabela Ratajczak, Daniel J. Chmura and Joanna Mucha

**Supplementary File S1**

**Common garden experiment – expanded data**

***The common garden establishment***

Common garden experiments are key to understanding local adaptation, allowing the evaluation of individual and combined traits across broad climatic gradients (Joyce and Rehfeldt, 2017; Sáenz-Romero et al., 2017). Thus, to evaluate the metabolic adaptation to new climatic condition, we used the common garden experiment established in spring 1967, testing 31 populations of Scots pine from Poland (Przybylski et al. 1968). Cones were collected in winter 1965/66 from local stands. The nativeness of each stand was determined through the consultation with local forest administration authorities before the collection of cones. Within a given stand, full randomness was adopted, meaning that no selection criteria other than cone bearing, were used when choosing trees (Przybylski, 1970). The collected cones from all provenances were transported to the Institute of Dendrology, Polish Academy of Sciences in Kórnik, where seeds were extracted. The seeds were processed without keeping identity of individual mother trees, as the main goal of this common garden experiment was to inventory the extent of among=population variation in quantitative traits within the species for the forestry practice, and its design was designated for this purpose (Przybylski, 1970).

In spring 1967 the experiment was planted in the Experimental Forest "Zwierzyniec" in Kórnik using 1-year-old seedlings. Each population was planted in eight, completely randomized blocks. In each plot, 49 seedlings representing a given provenance were planted in a 7×7 grid in the1.20 m × 0.50 m spacing, commonly used at that time for Scots pine (Przybylski 1970).

***Growth, survival and phenology of examined Scots pine KO and PPN provenances***

At the beginning of the common garden experiment, the survival percentage was assessed in (1971 and 1991 y. for 6- and 26-year-old trees) and was estimated on the level of 95.15 % and 21.48 % for KO and 88.27 % and 12.51 % for PPN, respectively (Fig. S1). In later years, the thinnings were made, inhibiting the further reliable analyses of survival rates.

Tree diameters (DBH – diameter at 1.3 m above the ground) of all living trees were measured in the experiment at ages 34 and 51 years. Subsequently, individual tree basal areas (BA based on DBH) were summed at the plot level and expressed per hectare basis. Although the average DBH per plot is only weakly dependent on the number of trees, the BA is strongly affected by stand density, and it better reflects the stand-level growth potential. The DBH, BA and survival at age 34 were assessed after the thinning, but both the early survival assessment at age 6, and further mortality after the thinning, indicate that the PPN provenance suffered higher mortality than the local KO provenance (Fig. S1), likely associated with the elimination of juvenile individuals least adjusted to the new environmental conditions.

Due to the metabolomic results suggesting a phenological shift between KO and PPN (see main manuscript), bud burst phenology of both provenances was observed at a single occasion in April 2025 using binoculars. Bud-burst was scored on all living trees in two examined populations (N=29 for KO, and N=21 for PPN) according to a scale ranging from 1-dormant bud to 4 – elongating bud with needles visible. This method has been successfully used do estimate the relative differences in phenology among Scots pine populations (Chmura et al. 2012). On average, the KO provenance showed significantly earlier bud burst phenology compared to the PPN provenance (Fig. S1).

**Fig. S1**. Growth traits, survival and bud-burst phenology of two examined provenances of Scots pine at the experimental site: Experimental forest ‘Zwierzyniec’ a) Tree diameter at 1.3 m above the ground, and b) basal area per ha at age 34 and 51 years; c) percent of living trees at ages 6, 34 and 51 years; d) bud burst phenology. Horizontal lines show average values across all tested provenances at given age. Thicker line within the box indicates median value, diamonds show mean values. Different lower-case letters indicate a statistically significant difference (Student t-test; α=0.05).

**References**

Chmura DJ, Rożkowski R, Chałupka W. 2012. Growth and phenology variation in progeny of Scots pine seed orchards and commercial seed stands', Eur. J. For. Res. 131: 1229-1243.

Joyce DG, Rehfeldt GE. 2017. Management strategies for black spruce (*Picea mariana* (Mill.) BSP) in the face of climate change: climatic niche, clines, climatypes, and seed transfer. Forestry 90(4):594-610.

Przybylski T. 1968. Metodyka doświadczeń proweniencyjnych z sosną zwyczajną (*Pinus sylvestris* L.) z terenu Polski, założonych przez Zakład Dendrologii i Arboretum Kórnickie PAN Arb Kórn 13:287-295.

Przybylski T. 1970. Zmienność sosny zwyczajnej (*Pinus sylvestris* L.). Zakład Dendrologii i Arboretum Kórnickie PAN w Kórniku. Habilitation thesis. pp. 61.

Sáenz‐Romero C, Lamy JB, Ducousso A, Musch B, Ehrenmann F, Delzon S, et al. 2017. Adaptive and plastic responses of *Quercus petraea* populations to climate across Europe. Global Change Biol. 23(7):2831-2847.
