# Supplementary material for: Provenance effect on the fine-root metabolome and structure of mature Scots pine revealed in a long-term common garden experiment
Source: Tree Physiol. 2026 Sep 8;46(10):tpag126. doi: 10.1093/treephys/tpag126 (PMC13652640; doi:10.1093/treephys/tpag126)
Supplement: Supplementary file 1 [file supplementary_materials_tpag126.zip › supplementary_materials_tpag126/Supplementary_Table_S1_Climatic_data_Minor_R_clear_copy_tpag126.docx]

**Provenance effect on the fine-root metabolome and structure of mature Scots pine revealed in a long-term common garden experiment**

Agnieszka Szuba, Pulak Maitra, Katarzyna Hrynkiewicz, Izabela Ratajczak, Daniel J. Chmura and Joanna Mucha

**Supplementary Table S1**

Geographic and climatic description of examined Scots pine populations and the planting site.

Climatic variables were derived from ClimateEU v.4.63 for populations and planting site for a period 1941-1970, corresponding to climate to which seed sources were adapted and the time of site establishment, and for 1991-2020 for planting site, corresponding to recent climate.

|  |  | PPN | Ko | site |  |  |
| --- | --- | --- | --- | --- | --- | --- |
| Latitude N |  | 49.41667 | 52.24447 | 52.24364 |  |  |
| Longidute E |  | 20.33333 | 17.07066 | 17.06739 |  |  |
|  |  | **PPN** | **Ko** | **site** | **site** |  |
|  |  | **Period** | | | |  |
| Variable | **Desciption** | **1941-1970** | | | **1991-2020** | **delta*** |
| MAT | mean annual temperature (°C) | 6.4 | 8.1 | 8 | 9.3 | 1.3 |
| MWMT | mean warmest month temperature (°C) | 16.6 | 18.1 | 18.1 | 19.2 | 1.1 |
| MCMT | mean coldest month temperature (°C) | -6.2 | -4 | -4 | -1.6 | 2.4 |
| TD | temperature difference between MWMT and MCMT | 22.8 | 22.1 | 22.1 | 20.8 | -1.3 |
| MAP | mean annual precipitation (mm) | 766 | 522 | 523 | 518 | -5 |
| MSP | mean summer (May to Sept.) precipitation (mm) | 458 | 292 | 292 | 293 | 1 |
| AHM | annual heat:moisture index | 21.4 | 34.7 | 34.4 | 37.3 | 2.9 |
| SHM | summer heat:moisture index | 36.2 | 61.9 | 61.9 | 65.4 | 3.5 |
| DD<0 | degree-days below 0°C, chilling degree-days | 563 | 386 | 388 | 228 | -160 |
| DD>5 | degree-days above 5°C, growing degree-days | 1689 | 2025 | 2018 | 2200 | 182 |
| DD<18 | degree-days below 18°C, cooling degree-days | 4275 | 3673 | 3683 | 3311 | -372 |
| DD>18 | degree-days above 18°C, heating degree-days | 52 | 148 | 145 | 217 | 72 |
| NFFD | the number of frost-free days | 213 | 243 | 243 | 263 | 20 |
| bFFP | the Julian date on which FFP begins | 117 | 108 | 108 | 102 | -6 |
| eFFP | the Julian date on which FFP ends | 279 | 293 | 293 | 297 | 4 |
| FFP | frost-free period | 162 | 185 | 185 | 195 | 10 |
| PAS | precipitation as snow (mm) between August in previous year and July in current year | 111 | 59 | 59 | 35 | -24 |
| EMNT | extreme minimum temperature over 30 years | -33.1 | -28.5 | -28.5 | -24.8 | 3.7 |
| Eref | Hargreaves reference evaporation | 629 | 614 | 613 | 659 | 46 |
| CMD | Hargreaves climatic moisture deficit | 44 | 218 | 217 | 246 | 29 |
| Tmax_wt | winter mean maximum temperature (°C) | -0.7 | 0.4 | 0.3 | 2.2 | 1.9 |
| Tmax_sp | spring mean maximum temperature (°C) | 11.4 | 12.8 | 12.8 | 14.4 | 1.6 |
| Tmax_sm | summer mean maximum temperature (°C) | 21.5 | 23.1 | 23 | 24.2 | 1.2 |
| Tmax_at | autumn mean maximum temperature (°C) | 12.5 | 13 | 13 | 13.5 | 0.5 |
| Tmin_wt | winter mean minimum temperature (°C) | -8.1 | -5.1 | -5.1 | -3.1 | 2 |
| Tmin_sp | spring mean minimum temperature (°C) | 1.4 | 3.1 | 3.1 | 4.3 | 1.2 |
| Tmin_sm | summer mean minimum temperature (°C) | 10.1 | 12 | 12 | 12.9 | 0.9 |
| Tmin_at | autumn mean minimum temperature (°C) | 2.8 | 5.2 | 5.1 | 5.7 | 0.6 |
| Tave_wt | winter mean temperature (°C) | -4.4 | -2.4 | -2.4 | -0.4 | 2 |
| Tave_sp | spring mean temperature (°C) | 6.4 | 8 | 7.9 | 9.3 | 1.4 |
| Tave_sm | summer mean temperature (°C) | 15.8 | 17.5 | 17.5 | 18.5 | 1 |
| Tave_at | autumn mean temperature (°C) | 7.7 | 9.1 | 9.1 | 9.6 | 0.5 |
| PPT_wt | winter precipitation (mm) | 113 | 95 | 95 | 94 | -1 |
| PPT_sp | spring precipitation (mm) | 170 | 114 | 114 | 111 | -3 |
| PPT_sm | summer precipitation (mm) | 328 | 202 | 202 | 200 | -2 |
| PPT_at | autumn precipitation (mm) | 155 | 111 | 111 | 113 | 2 |

* delta shows the difference between 1941-1970 and 1991-2020 periods at planting site
